# Determining optimal Barthel Index cutoff scores for predicting Longshi Scale grades across age groups in stroke patients

**DOI:** 10.3389/fragi.2026.1701910

**Published:** 2026-02-09

**Authors:** Jing Zhang, Mingchao Zhou, Ankang Liu, Ruixue Ye, Yulong Wang

**Affiliations:** 1 Department of Rehabilitation, Shenzhen Second People’s Hospital, First Affiliated Hospital of Shenzhen University, Shenzhen, Guangdong, China; 2 Department of Nursing, Shenzhen Dapeng New District Nan’ao People’s Hospital, Shenzhen, Guangdong, China

**Keywords:** activities of daily living, Barthel index, cutoff score, disability, receiver operating characteristic curve

## Abstract

**Background:**

The Barthel Index (BI) is a standard, widely used measure of dependence in activities of daily living (ADL), particularly in stroke care. The Longshi Scale (LS) offers a simpler, more user-friendly alternative; however, it lacks a validated, age-stratified mapping to BI scores. This gap limits consistent outcome interpretation and application.

**Objective:**

This study aims to establish and validate a standardized, age-stratified concordance between BI scores and LS grades, thereby providing a practical conversion tool for clinical and research settings.

**Method:**

In a multi-center study of 16,412 stroke inpatients (3 months post-stroke), BI scores and LS grades were analyzed across age groups: <60 years (n = 12,662), 60–79 years (n = 2,596), and ≥80 years (n = 1,154). Sensitivity (correct identification) and specificity (correct exclusion), along with receiver operating characteristic (ROC) curves were used to determine optimal BI cutoff points for each LS grade. Spearman correlation and the Kruskal–Wallis test were applied across age groups.

**Results:**

Key BI cutoffs were identified for LS grades: ≥75 for LS ≥ 2, ≥45 for LS ≥ 4, and <5 for LS = 6. These cutoff values were consistent across age groups. The BI scores were negatively correlated with LS disability level (e.g., r = −0.879 in patients aged ≥80 years, *p* < 0.001). Correlations remained strongest at severe disability levels (LS grades 5–6) across age groups (r = −0.60 to −0.65). AUC analysis demonstrated excellent discriminative ability, particularly for the mildest (LS 1) and most severe (LS 6) disability levels (AUC >0.95).

**Conclusion:**

The study provides age-stratified BI cutoff values to guide resource allocation, emphasizing the need to prioritize care for individuals aged ≥80 years with BI scores below 5.

## Introduction

Stroke is a leading global cause of long-term disability, affecting approximately 15 million people annually, with one-third (5 million) left permanently disabled, which results in dependency (GBD 2021 Stroke Risk Factor Collaborators, 2024; Zandvliet et al., 2019; Hotter et al., 2018; Shin et al., 2022). Assessment of functional independence is critical for measuring post-stroke outcomes (Littooij et al., 2022). The Barthel Index (BI), widely adopted globally, evaluates rehabilitation across diverse populations but faces limitations in its classification, which inadequately addresses age-related functional heterogeneity in complex clinical scenarios (Zhang et al., 2021). Alternatively, the Longshi Scale (LS), a clinically based tool ranging from complete independence (LS grade 1) to complete dependence (LS grade 6), provides granular disability assessment, particularly for sensory/communication domains, although it remains underutilized outside China, particularly in the long-term care of disabled patients (Chamberlain et al., 2016; Wang et al., 2020). Collectively, these metrics constitute critical indicators for assessing post-stroke dependence in activities of daily living (ADL). BI provides a fine-grained score useful for detailed tracking and research, while the LS offers a rapid, categorical assessment advantageous in busy clinical settings or for triage. Although both BI and LS assess post-stroke disability, discrepancies in their scoring frameworks cause interpretive confusion (Liu et al., 2022). Crucially, aging populations exhibit distinct functional trajectories, yet the interplay between age, BI scores, and LS grades remains unquantified.

At present, translated BI versions in multiple translations provide a reliable measure of basic ADL for evaluating the effectiveness of rehabilitation. Aging populations demand increasingly precise long-term care, yet BI’s broad categories may obscure age-specific functional declines (e.g., slower recovery in elderly patients). In comparison, the LS is a clinically based evaluation tool for disability that comprises six grades (Lübke et al., 2004; Pan et al., 2021). LS’s contextual sensitivity might better capture age-dependent care needs (e.g., domestic vs. bedridden states in older adults), particularly in emotional communication such as visual, auditory, and speech functions. Although it has been used frequently in large-scale outcome studies, it remains underutilized outside China (Zhang et al., 2024). Current studies lack consensus on the correspondence between BI–LS and age stratification, impeding personalized rehabilitation and cross-study comparisons (Liu et al., 2022; Zhou et al., 2021; Mueller-Schotte et al., 2020). This gap is critical given global demographic shifts toward older stroke populations.

Clinically defining LS grades and BI scores in stroke patients is an important issue not only for evaluating individual patient outcomes but also for evaluating the classification of disability levels among people of different age groups (Gao et al., 2021). It has been argued that LS can be utilized in conjunction with the BI for a comprehensive assessment to reflect the degree of disabilities comprehensively (Zhou et al., 2023). Disability measured by the LS and ADL measured by the BI often reveal discrepancies in explaining the functional outcomes of stroke survivors (Liu et al., 2023). Thus, this study aims to establish optimal age-stratified BI cutoffs for LS grades and support the design of clinical trials that utilize both measures. Understanding the BI–LS relationship across LS grades was intended to differentiate clinically distinct disability grades, thereby revealing the correspondence between the LS grades and BI scores and enabling precise translation between scales for tailored, age-informed clinical decision-making in stroke survivors.

## Materials and methods

### Study design and participants

This prospective multi-center project investigates residual disabilities, activity limitations, and the degree of disability in patients after stroke. Patients were identified and assessed during the post-acute, stable phase after stroke onset. All eligible patients reaching this assessment window were recruited between August 2022 and May 2023. Informed consent was obtained online from all participants prior to enrollment. If a patient was unable to provide informed consent due to incapacity, consent was obtained from their legally authorized representative.

### Inclusion and exclusion criteria

#### Inclusion criteria

The ***inclusion*** criteria are as follows:

- Aged ≥18 years
- Diagnosed with ischemic or hemorrhagic stroke according to WHO criteria
- Stroke onset approximately 3 months (i.e., 12–16 weeks) prior to the assessment
- Voluntarily provided signed informed consent.

#### Exclusion criteria

The exclusion ***criteria*** are as follows:

- Comorbidities affecting functional status (e.g., fractures or amputations)
- Occurrence of serious adverse events, poor compliance, or inability to complete assessments during the study
- Critical illness precluding participation in rehabilitation interventions

### Evaluation tool

***The Barthel Index is*** a validated 100-point scale comprising 10 ADL items, including feeding, bathing, grooming, dressing, bowel and bladder control, toilet use, transfers (e.g., bed to chair), mobility, and stair climbing. The scores for items related to lower and upper extremity function, such as mobility and feeding, respectively, are particularly indicative of motor recovery (Yan et al., 2024). Each item is graded on a three-tiered system reflecting the degree of physical assistance required. This internationally recognized instrument, including its multilingual adaptations, demonstrates high reliability in assessing rehabilitation outcomes and has been extensively implemented in clinical trials and observational studies (Collin et al., 1988; Winstein et al., 2016).

**FIGURE 1:**
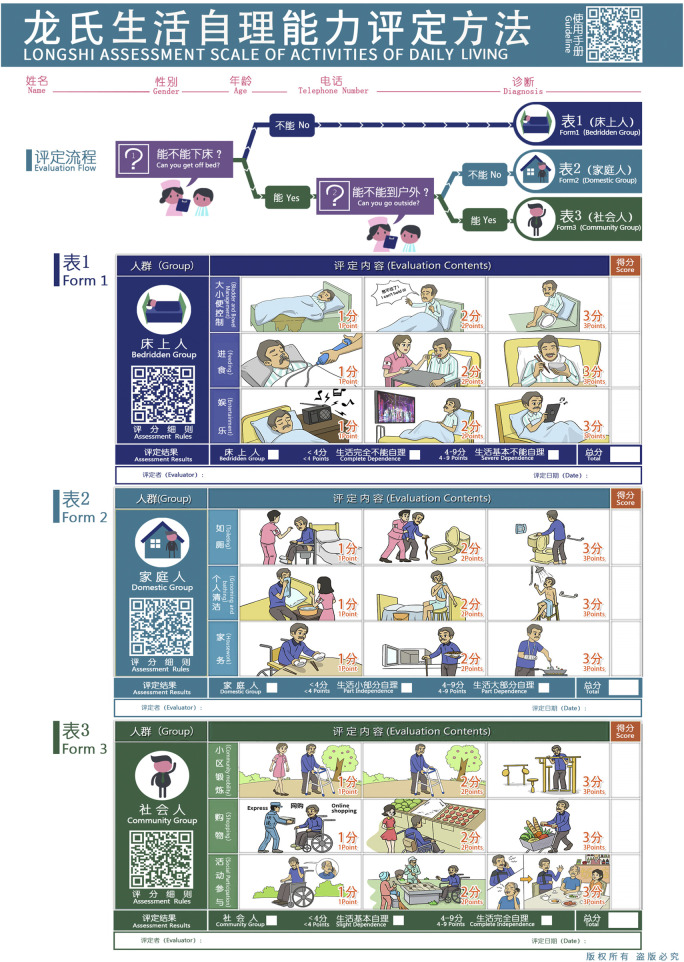
Longshi Scale for assessing the activities of daily living. (Reproduced from Liu et al., 2022, under the terms of the Creative Commons Attribution 4.0 International License).

**FIGURE 2:**
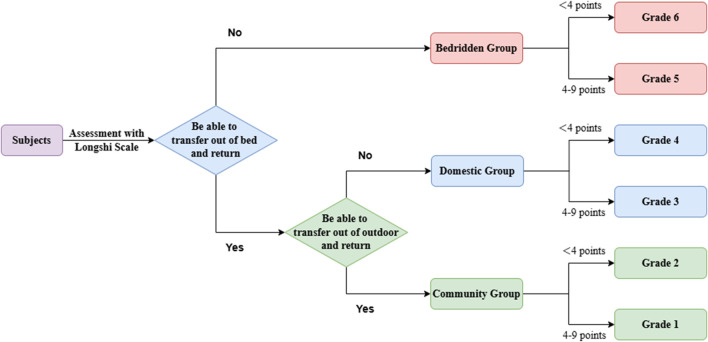
Assessment process for disability grades. LS: Grade 1, complete independence; Grade 2, slight dependence; Grade 3, most self-care; Grade 4, partial self-care; Grade 5, severe dependence; Grade 6, complete dependence.

***The Longshi Scale*** is a scientifically validated tool that categorizes individuals into bedridden, domestic, and community groups based on their mobility (Liu et al., 2020). It further classifies patients into six disability grades, from Grade 1 (complete independence) to Grade 6 (complete dependence). Recommended as a national standard in 2018, this scale assesses disability using three key groups and six grades (Bahlman-van Ooijen et al., 2025), as shown in Figure 1. The assessment process for disability grades is shown in Figure 2. LS grades 5 and 6 fall within the bedridden group, with grade 6 representing the most severe level, in which patients are unable to get out of bed independently and can only move within the bed. LS grades 3 and 4 belong to the domestic group, in which patients can get out of bed and move independently within the home but are limited to indoor activities. LS grades 1 and 2 belong to the community group, in which patients are able to transfer outdoors and engage in outdoor activities (including using a wheelchair) (Xiong et al., 2023).

### Data collection

The BI scores and LS grades were assessed using the Barthel Index and Longshi Scale through face-to-face interviews conducted by evaluators with the patients. To ensure optimal validity and interrater reliability, all data collection via questionnaires and patient evaluations, including the LS and BI, were performed by healthcare professionals, including physicians, nurses, and therapists, who received online training and understood the purpose of the study. Demographic variables and clinical characteristics were collected through structured electronic questionnaires administered via a secure WeChat-based platform (Mini Program). Several trained professional assessors independently evaluated participants using these standardized electronic questionnaires. Assessments were conducted during the stable post-stroke phase (approximately 3 months), with research coordinators at each center completing data collection within this time window.

### Analysis methods

Statistical analyses were performed using the software package SPSS 24.0 (SPSS Inc., Chicago, IL, United States). The normality of continuous data distribution was assessed using the Shapiro–Wilk test. A significance level of *p* < 0.05 was set to reject the null hypothesis of normality (Benjamin et al., 2018). Because the data did not satisfy the assumption of normal distribution, all statistical tests applied were nonparametric. Based on the normality assessment, continuous variables are presented as the mean ± standard deviation (if normally distributed) or the median and interquartile range (if non-normally distributed). Categorical variables are presented as counts and percentages. Descriptive statistics were used to examine the distribution of the BI scores according to the LS grade. Sensitivity refers to the proportion of patients correctly identified by the BI cutoff for a given LS grade, while specificity refers to the proportion correctly excluded. To determine appropriate BI cutoff values corresponding to each LS grade, we calculated the sensitivity and specificity of multiple BI scores for discriminating between adjacent LS grades (e.g., grade 2 vs. grade 3). The score that maximized the Youden index (J = sensitivity + specificity − 1) was selected as the optimal cutoff. This method balances the correct identification of patients within a target LS grade (sensitivity) and the correct exclusion of those not in that grade (specificity).

Positive predictive value (PPV) represents the probability that a patient with a BI score above the cutoff truly belongs to the higher LS grade. Negative predictive value (NPV) represents the probability that a patient with a BI score below the cutoff truly belongs to the lower LS grade. The strength of the correlation was interpreted as follows: |r| <0.3 indicated a weak correlation; 0.3 ≤ |r| <0.7 indicated a moderate correlation; and |r| ≥0.7 indicated a strong correlation (Cohen, 2013). Spearman correlation analysis was used to examine the relationship between LS grades and BI scores, and the Kruskal–Wallis H test was used to compare BI scores across different LS grades. Where a significant difference was found, *post hoc* pairwise comparisons were conducted using Dunn’s test, applying a Bonferroni correction for multiple comparisons. The effect size for the Kruskal–Wallis test was calculated as eta-squared (η^2^) based on the H statistic, with values interpreted as follows: 0.01, small; 0.06, medium; and 0.14, large.

Receiver operating characteristic (ROC) curve analysis was employed to determine the optimal BI cutoff scores for discriminating adjacent disability levels defined by the LS. Given the ordinal nature of the LS, a threshold-based approach was utilized. The optimal cutoff point on the ROC curve for each comparison was selected by maximizing the Youden index (J = sensitivity + specificity − 1), which identifies the value that best balances the trade-off between sensitivity and specificity.

## Results

### Population characteristics

**FIGURE 3:**
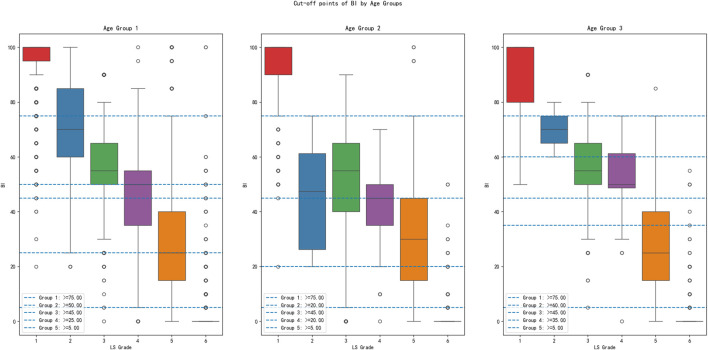
Distribution of BI scores within the LS grades across age groups.

The patients’ baseline characteristics are shown in Table 1. Of the 16,412 patients, 6,387 (38.9%) were male and 10,025 (61.1%) were female. In addition, 12,662 (77.2%) were younger than 60 years, 2,596 (15.8%) were between the ages of 60 and 79, and 1,154 (7.0%) were ≥80 years. Figure 3 illustrates the BI frequency distribution in each LS grade and demonstrates the median, inter-quartile range, minimum, and maximum of the BI scores.

**TABLE 1:** Baseline characteristics of LS grades recorded based on age groups.

Age group	LS grade	Patient count, n (%)	Male, n (%)	Female, n (%)	BI score, median (IQR)
<60	LS_Grade 1	4,325 (34.2)	1,690 (35.9)	2,635 (33.1)	100 [95.00, 100.00]
​	LS_Grade 2	72 (0.6)	24 (0.5)	48 (0.6)	70 [60.00, 85.00]
​	LS_Grade 3	2,756 (21.8)	1,039 (22.1)	1717 (21.6)	55 [50.00, 65.00]
​	LS_Grade 4	496 (3.9)	185 (3.9)	311 (3.9)	50 [35.00, 55.00]
​	LS_Grade 5	3,393 (26.8)	1,163 (24.7)	2,230 (28.0)	25 [15.00, 40.00]
​	LS_Grade 6	1,620 (12.8)	605 (12.9)	1,015 (12.8)	0 [0.00, 0.00]
60–79	LS_Grade 1	592 (22.8)	255 (20.6)	337 (24.8)	100 [90.00, 100.00]
​	LS_Grade 2	6 (0.2)	5 (0.4)	1 (0.1)	47.5 [26.25, 61.25]
​	LS_Grade 3	1,042 (40.1)	444 (35.9)	598 (44.0)	55 [40.00, 65.00]
​	LS_Grade 4	77 (3.0)	35 (2.8)	42 (3.1)	45 [35.00, 50.00]
​	LS_Grade 5	575 (22.1)	326 (26.4)	249 (18.3)	30 [15.00, 45.00]
​	LS_Grade 6	304 (11.7)	171 (13.8)	133 (9.8)	0 [0.00, 0.00]
≥80	LS_Grade 1	155 (13.4)	37 (8.3)	118 (16.6)	100 [80.00, 100.00]
​	LS_Grade 2	2 (0.2)	1 (0.2)	1 (0.1)	70 [65.00, 75.00]
​	LS_Grade 3	311 (26.9)	144 (32.4)	167 (23.6)	55 [50.00, 65.00]
​	LS_Grade 4	44 (3.8)	13 (2.9)	31 (4.4)	50 [48.75, 61.25]
​	LS_Grade 5	373 (32.3)	130 (29.2)	243 (34.3)	25 [15.00, 40.00]
​	LS_Grade 6	269 (23.3)	120 (27.0)	149 (21.0)	0 [0.00, 0.00]
Total	LS_Grade 1	5,072 (30.9%)	1982 (31.0%)	3,090 (30.8%)	100 [95.00, 100.00]
​	LS_Grade 2	80 (0.5%)	30 (0.5%)	50 (0.5%)	70 [55.00, 80.00]
​	LS_Grade 3	4,109 (25.0%)	1,627 (25.5%)	2,482 (24.8%)	55 [50.00, 65.00]
​	LS_Grade 4	617 (3.8%)	233 (3.6%)	384 (3.8%)	50 [35.00, 55.00]
​	LS_Grade 5	4,341 (26.4%)	1,619 (25.3%)	2,722 (27.2%)	25 [15.00, 40.00]
​	LS_Grade 6	2,193 (13.4%)	896 (14.0%)	1,297 (12.9%)	0 [0.00, 0.00]

LS, Longshi Scale.

SD, standard deviation.

BI, Barthel Index.

The distribution of BI total scores varied across the different LS grades, as visually summarized in Figure 3. Lower LS grades (indicating greater dependence) were associated with a clustering of higher BI scores, while higher LS grades showed a wider spread and lower median BI scores.

## Optimal BI cutoff scores for LS grades

The optimal cutoff score of the BI was determined to distinguish different disability grades of the LS. The sensitivity, specificity, PPV, and NPV for each age group and the total population are detailed in Table 2.

The BI score effectively distinguishes between different LS grades, with optimal cutoff values varying by both age and the specific LS grade being distinguished. Table 2 presents the optimal BI cutoff scores for distinguishing different levels of disability severity. Furthermore, when examining different age groups, certain differences in the BI cutoff values were observed. Each cutoff value corresponds to the level at which reaching or exceeding this score classifies the patient into this LS grade or a lower numerical grade (indicating better functionality). The Youden index values of LS Grade 1 were 0.903, 0.858, and 0.850, respectively, across age groups (<60 years, 60–79 years, and ≥80 years). The results for LS Grade 1 and LS Grade 6 confirm that BI ≥75 and BI < 5 are reliable criteria for identifying patients with complete independence and complete dependence, respectively. However, the model’s low PPV for LS Grade ≥3 suggests that BI cutoff scores alone lack the precision to cleanly differentiate between adjacent levels of moderate dependency as they result in a high number of false positives.

**TABLE 2:** BI cutoff scores corresponding to LS grades.

Age group	LS group	BI cutoff score	Sensitivity	Specificity	Youden index*	PPV	NPV
<60	LS grade ≥2	≥75	0.966	0.937	0.903	0.888	0.982
​	LS grade ≥3	≥50	0.917	0.414	0.331	0.009	0.999
​	LS grade ≥4	≥45	0.952	0.447	0.399	0.324	0.971
​	LS grade ≥5	≥25	0.915	0.247	0.162	0.047	0.986
​	LS grade = 6	<5	0.916	0.151	0.067	0.283	0.830
60–79	LS grade ≥2	≥75	0.922	0.936	0.858	0.810	0.976
​	LS grade ≥3	≥20	1.000	0.220	0.220	0.003	1.000
​	LS grade ≥4	≥45	0.746	0.481	0.227	0.491	0.738
​	LS grade ≥5	≥20	0.948	0.224	0.172	0.036	0.993
​	LS grade = 6	<5	0.910	0.161	0.071	0.236	0.862
≥80	LS grade ≥2	≥75	0.884	0.966	0.850	0.806	0.981
​	LS grade ≥3	≥60	1.000	0.700	0.700	0.006	1.000
​	LS grade ≥4	≥45	0.931	0.654	0.585	0.499	0.963
​	LS grade ≥5	≥35	0.909	0.457	0.366	0.063	0.992
​	LS grade = 6	<5	0.869	0.277	0.146	0.365	0.815
Total	LS grade ≥2	≥75	0.959	0.939	0.898	0.876	0.981
​	LS grade ≥3	≥50	0.888	0.429	0.317	0.008	0.999
​	LS grade ≥4	≥45	0.898	0.465	0.363	0.359	0.932
​	LS grade ≥5	≥25	0.916	0.258	0.174	0.046	0.987
​	LS grade = 6	<5	0.911	0.160	0.071	0.281	0.833

*Youden index = sensitivity + specificity – 1.

PPV, positive predictive value; NPV, negative predictive value; BI, Barthel Index; LS, Longshi Scale.

LS_Grade 6: not assigned a separate cutoff score, representing complete dependence (typically BI scores < cutoff for Grade 5).

BI cutoff score: A BI score ≥ this value suggests the patient is more likely to be classified in the higher of the two adjacent LS grades being compared (e.g., BI ≥ 50 for distinguishing LS grade 2 vs. 3).

## Correlation between LS grades and BI scores

The correlations between LS grades and BI scores were stratified by three age groups and are presented in Table 3. Further analysis of the BI's discriminatory power across the three overarching LS categories (Bedridden, Domestic, and Community) is provided in the Supplementary Material (Supplementary Figures 1–4).

**TABLE 3:** Correlation between LS grades and BI scores.

LS		BI					
LS group	LS grade	Age group 1 (<60)		Age group 2 (60–79)		Age group 3 (≥80)	
		r (*p*)	K–W test (*p*)	r (*p*)	K–W test (*p*)	r (*p*)	K–W test (*p*)
Community	LS_Grade 1	−0.326 (<0.001)	6.473 (<0.001)	−0.352 (<0.001)	3.378 (<0.001)	−0.179 (0.025)	0.556 (0.578)
​	LS_Grade 2	​	​	​	​	​	​
Domestic	LS_Grade 3	−0.331 (<0.001)	7.593 (<0.001)	−0.100 (<0.001)	2.726 (0.006)	−0.132 (0.013)	1.113 (0.266)
​	LS_Grade 4	​	​	​	​	​	​
Bedridden	LS_Grade 5	−0.600 (<0.001)	23.223 (<0.001)	−0.652 (<0.001)	11.644 (<0.001)	−0.604 (<0.001)	10.752 (<0.001)
​	LS_Grade 6	​	​	​	​	​	​
Total	​	−0.917 (<0.001)	​	−0.834 (<0.001)	​	−0.879 (<0.001)	​

r: Spearman correlation coefficient.

K–W test, Kruskal–Wallis test; Bonferroni-adjusted *p*-values remained significant for both subgroups.

Spearman correlation analysis revealed a strong negative association between BI scores and LS disability grades. Specifically, the strong negative correlation coefficients (r) were −0.917 in the <60 years group, −0.834 in the 60–79 years group, and −0.879 in the ≥80 years group (all *p* < 0.001). Within the bedridden subgroup (LS grades 4 and 5, representing moderate-to-severe and severe dependence, respectively), this negative correlation remained moderately strong, with r values ranging from −0.652 to −0.600 across age groups (all *p* < 0.001).

## AUC for the BI cutoff score

The area under the curve (AUC) and the corresponding optimal BI cutoff scores derived from ROC analysis for each age group are summarized in Table 4.

**TABLE 4:** AUC corresponding to LS grades among different age groups.

LS_ Grade	Area under the curve (AUC)				SE	Asymptotic significance	Asymptotic 95% confidence interval	
	Age group 1	Age group 2	Age group 3	Total			Lower bound	Upper bound
1	0.99	0.97	0.98	0.96	0.0014	<0.0001	0.9590	0.9643
2	0.76	0.68	0.68	0.76	0.0160	<0.0001	0.5392	0.6050
3	0.91	0.81	0.89	0.84	0.0042	<0.0001	0.5640	0.5809
4	0.77	0.77	0.74	0.82	0.0052	<0.0001	0.5854	0.6072
5	0.90	0.83	0.83	0.90	0.0036	<0.0001	0.7728	0.7869
6	0.97	0.97	0.95	0.98	0.0014	<0.0001	0.9690	0.9744

**FIGURE 4:**
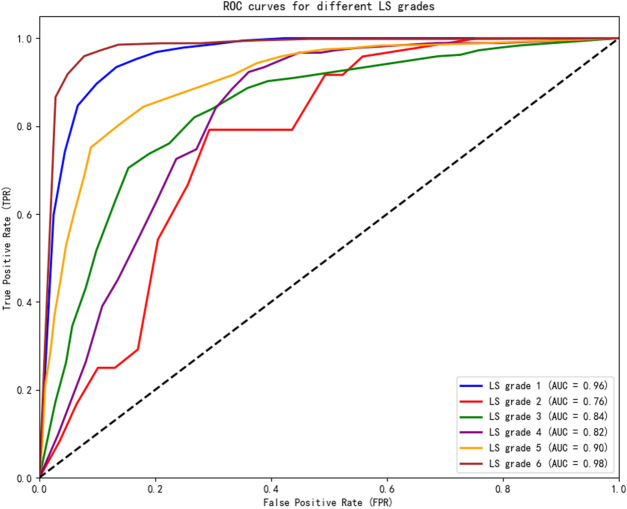
ROC curves of BI cutoff scores corresponding to LS grades.

All AUC values were statistically significant (greater than 0.5, *p* < 0.05). Notably, AUC values exceeding 0.9 for several key distinctions indicate excellent diagnostic accuracy. These results are further illustrated in the ROC curves shown in Figure 4.

As shown in Figure 4, AUC values were higher than 0.90 for LS levels 1 and 6, with the highest for LS grade 6 (AUC = 0.98), indicating that the BI has excellent discriminative ability for these grades.

## Discussion

### Optimal cutoff scores and diagnostic performance of BI for LS grades

In this study, we analyzed the statistically significant association between LS and BI by calculating the optimal BI cutoff scores for each LS grade. Based on our results, the optimal BI cutoffs for the total cohort and the <60-year age subgroup were identified as follows: ≥75 distinguishing LS Grade 1 from LS Grade ≥2), ≥50 distinguishing LS Grade ≤2 from LS Grade ≥3, ≥45 distinguishing LS Grade ≤3 from LS Grade ≥4, ≥25 distinguishing LS Grade ≤4 from LS Grade ≥5, and <5 identifying completely dependent patients (LS Grade 6). Notably, the BI cutoffs of ≥75 and <5 demonstrate exceptionally high discriminatory power by clearly separating the clinically distinct states of “complete independence” (LS Grade 1) and “complete dependence” (LS Grade 6). This moderate performance for intermediate grades (e.g., 2 and 4) aligns with the fact that the functional transitions between these adjacent LS levels (e.g., from moderate to mild dependence) are behaviorally more subtle and complex, making them inherently more challenging for a global functional measure like the BI to delineate sharply (Gittler and Davis, 2018). To our knowledge, this is the first study to establish BI cutoff scores corresponding to LS grades across age groups in stroke patients. The BI provides a multidimensional assessment of basic ADL and is increasingly recognized as a primary foundation for designing informal care interventions for individuals with disabilities (Bahlman-van Ooijen et al., 2025). The findings of this study may contribute to the evaluation of LS grades. For example, changes in LS grades could help determine clinically meaningful improvements in ADL function among stroke patients. Although there is currently no unified dichotomization for the LS grades, this study aims to establish and validate data-driven cutoffs to address this gap.

When evaluating the LS grade, it is clear that a BI score of 75 or above is classified as LS 1 (complete independence/slight dependence), and a BI score of less than 5 is classified as LS 6 (severe dependence/complete dependence). For patients with complete disability, the BI cutoff is highly consistent with other studies and is relatively accurate. The BI, with its finer granularity (0–100 scale), is more sensitive to small, incremental changes in function, making it superior for detailed progress tracking in rehabilitation research or intensive therapy settings and for predicting specific outcomes such as length of stay. Conversely, the LS, with its categorical grades, excels in rapid triage in busy clinical environments (e.g., emergency departments and outpatient clinics), facilitating quick decision-making about care pathways and in large-scale population screening or registry work, where speed and simplicity are paramount. The BI provides depth, while the LS provides efficiency. The combination of LS and BI can address specific application problems in scenarios, particularly in determining LS 6 grade when BI <5. For patients with extremely low BI scores (ADL ability grade 0), the BI may have difficulty distinguishing subtle differences in functional ability. However, the BI cutoff score addresses the “floor effect” of BI by providing a crucial stratification within the conventionally defined “severe disability” band (BI <40), thus may help distinguish the differences in daily living activities between completely disabled patients and those with other LS grades in care scenarios (Sahin and Acaröz, 2025; Balu, 2009). Although BI has been considered sufficient for measuring physical function in elderly rehabilitation patients, it may lack sensitivity for detecting finer, clinically meaningful gradations of disability, particularly in stroke populations (Quinn et al., 2011). Given that BI originates from and is strongly correlated with ADL, the cutoff values in LS 1–6 can help caregivers identify patients in LS grade 6 who are completely dependent, thereby further formulating care plans. For instance, a patient classified as LS grade 3 (moderate dependence) with a BI score of 60 may have specific deficits in stair climbing and bathing. This detailed BI profile, interpreted within the broader functional category provided by the LS, allows the clinical team to formulate a targeted care plan focusing on lower limb strength training and adaptive equipment for bathing while also setting a realistic goal of progressing toward LS grade 4 (mild dependence).

### Differences in BI cutoff scores across LS grades among different age groups

The analysis revealed that several critical BI cutoffs remained consistent across age groups, specifically ≥75, ≥45, and <5. The optimal BI cutoff scores for distinguishing between adjacent LS grades did not show significant variation when analyses were conducted across different age groups in our study. This suggests that these cutoffs are robust and potentially generalizable across these subpopulations, reinforcing their utility as stable benchmarks for functional classification. In terms of age-stratified patterns in LS grades, while the overall inverse relationship between BI scores and LS grades remained consistent across age groups, significant intergroup variations emerged, necessitating age-specific interpretation. Critically, the cutoffs for the ≥80-year-old individuals were found to be higher than those of other age groups. From the study results, an age-specific pattern is evident: the proportion of patients with mild dependence (Grade 1) in the ≥80-year-old group sharply decreases, while over 55.6% fall into the severe dependence categories (grades 5–6). This aligns with real-world observations of a high degree of disability among the elderly. Therefore, this study suggests a dual-focused resource allocation strategy. Substantial resources must be directed to support and care for those with severe dependence (LS grades 5–6). Concurrently, the significant proportion of patients in the mild-to-moderate dependence categories (LS grades 1–4) presents a critical opportunity for secondary prevention and early intervention. Community-based initiatives such as tailored exercise programs, fall prevention strategies, and self-management education aimed at these individuals could help maintain functional capacity, reduce the risk of transitioning to higher dependence categories, and ultimately lessen the long-term societal care burden. This approach advocates for a more comprehensive and stratified allocation of healthcare resources (Doi et al., 2020).

The optimal BI cutoffs demonstrated marked age dependence, reflected in the composite sum score (sensitivity + specificity). The sum score for determining cutoff values reflects the optimal balance between maximizing the ability to correctly identify LS grades. Although BI has high discrimination efficacy for LS Grade 1 (0.898), we observe a slight downward trend in this index in the elderly group (from 0.903 in the <60-year-old group to 0.850 in the ≥80-year-old group). This may be due to comorbidities or age-related frailty, which makes the BI score pattern of elderly patients more heterogeneous, even though they are clinically assessed at the ‘community activity’ level (Bouwstra et al., 2019). It is notable that BI exhibited the weakest correlation coefficient for LS Grades 2–4 in the ≥80 group, indicating that the correlation shifts with age. In LS grades 5 and 6, the correlation between LS and BI is strongest in age group 3 (≥80 years old) compared with groups 1 and 2 (<60 years old and 60–79 years old, respectively) among different age groups. This indicates that, for the elderly, combining BI cutoff scores may be necessary to most accurately define their functional status.”

An interesting finding of this study is that sequential BI declines across LS grades 1–6 in the age group 1 (<60 years). For the age group 2 (60–79 years), there is substantial BI overlap in moderate grades (LS 2–5). In addition, for the age group 3 (≥80 years), there is pronounced merging of BI ranges between LS grades 3–4. The distribution range of BI cutoff values varies among LS grades representing different age groups. BI provides a limited ability to identify disability grades for elderly patients, especially in the moderate LS grades, and the cutoff values need to be combined for determination.

### Interplay between the LS and BI in stroke disability assessment

The differential performance of the LS and BI stems from their inherent design philosophies. The LS prioritizes dysfunction, particularly mobility, often leading to a classification of “bedridden” (LS Grade 6) for patients with severe movement impairment. In contrast, the BI is a multi-item ADL score that credits preserved function in non-mobility tasks. Consequently, a patient with stroke might be classified as LS Grade 6 yet achieve a moderate or even high BI score by retaining the ability to perform self-care activities (e.g., eating and grooming) in bed with minimal assistance. This fundamental discrepancy explains seemingly paradoxical clinical observations and underscores why the BI may have limited distinguishing power for functional status in patients with moderate, mobility-defined disabilities. Therefore, we recommend a combined or sequential use of both scales in clinical practice and research. The LS can serve as an efficient tool for initial screening and gross categorization, while the BI can be deployed for detailed assessment, progress monitoring, and nuanced outcome measurement in selected patients or research protocols.

For older adults, particularly those aged ≥80 years, BI scores should be interpreted within an age-specific context and integrated with complementary assessments to minimize the risk of misclassification. Accordingly, resource allocation strategies should reflect this functional gradient, with particular emphasis on the ≥80-year-old age group, among whom more than 55% exhibited severe functional dependence. This interpretation is supported by previous studies, which have proposed a BI threshold of ≤60 at discharge to define new-onset functional impairment. Furthermore, studies combining the BI with discriminant function analysis have demonstrated concordance with standard expert judgment in over 86% of nursing status classifications. Importantly, the remaining discrepancies were predominantly observed in borderline cases between adjacent levels of nursing care (Masuda et al., 2024; Maidhof et al., 1999). For stroke patients with mild to severe disability, the LS has demonstrated higher responsiveness compared to the BI, potentially due to the ceiling and floor effects of the BI (Gao et al., 2023). Similarly, the BI is less precise than the LS in capturing overall changes in disability status, particularly in aspects such as emotional communication and recreational activities. The LS includes items that assess emotional wellbeing and social interaction, areas that are often overlooked in functional status evaluations using the BI. These domains are vital to functional recovery, especially in elderly patients with moderate disability, where traditional measures such as the BI may fail to detect significant declines in social and emotional functioning (Wang et al., 2019). Importantly, a maximum BI score of 100 (indicating independence in all 10 items) does not necessarily imply independence in more detailed ADLs (Bahlman-van Ooijen et al., 2025).

These findings collectively suggest that the BI alone may be insufficient for detecting subtle changes in functional impairment, particularly among patients with mild stroke. Nevertheless, the AUC values in this study were generally above 0.8 (except for LS Grade 2), confirming that the BI provides theoretical support for the scientific assessment of LS grades as a quantitative measure of functional independence in ADLs.

## Implications of assessment for disabled individuals

Currently, most clinical applications use the BI scale. Our team independently developed the national standard LS earlier, which aligns with BI and demonstrates high consistency, reliability, and validity in assessing disability levels (Wang et al., 2019; Zhou et al., 2024). The precise correspondence between BI cutoffs and LS grades provides a quantitative tool for clinical practice, reducing reliance on imaging exams while enabling quick disability assessments.

The LS possesses distinct Chinese characteristics and offers a clinically efficient alternative to conventional disability assessments, with its pictorial-based design enabling rapid evaluation without specialized training (Wang et al., 2019; Liu et al., 2023). This research provides critical benchmarks for clinical practice: a BI score ≥75 serves as a reliable indicator of functional independence, while a score <5 flags complete dependency, necessitating prioritized care. Importantly, assessments for adults aged ≥80 must account for their higher disability threshold, where a seemingly moderate BI score may still conceal significant care needs. These findings support the combined use of BI scores and LS grades to provide a nuanced and comprehensive assessment of functional status. For instance, to provide a comprehensive assessment for older adults requiring care across different age groups, rather than relying solely on the BI (Ohura et al., 2014).

## Strengths and limitations

The LS offers practical advantages due to its simplicity and minimal resource requirements, enhancing its feasibility for large-scale studies (Xue et al., 2024). Our validation of precise BI–LS cutoffs provides statistically robust criteria for disability grading, which is particularly valuable in resource-limited settings. However, this study has several limitations. First, as participants were recruited from inpatient rehabilitation units, the sample may over-represent survivors with rehabilitation potential and under-represent those with the most severe strokes, introducing selection bias. Nonetheless, this setting was necessary to obtain the large volume of paired BI and LS assessments required for robust cutoff determination. Second, the validity of the identified cutoff scores needs to be confirmed in more diverse populations, including non-Chinese ethnic groups and patients with disabilities from other etiologies. Finally, readers should note that the cutoff for Grade 2 in the 60–79 age group was based on a limited subsample (n = 6). While presented for completeness, this value should be considered preliminary and interpreted cautiously until validated in larger cohorts.

## Conclusion

This study establishes and validates a critical bridge between the Barthel Index and the Longshi Scale by defining optimal, age-stratified cutoff scores that map BI scores to distinct LS disability grades. Our findings demonstrate that the BI excellently discriminates the extremes of functional independence (LS Grade 1) and complete dependence (LS Grade 6), while its performance for intermediate grades is less pronounced.

The BI cutoffs provide a standardized, quantitative method to enhance the precision of disability assessment in clinical practice, particularly within the conventionally poorly differentiated “severe disability” band (BI <40). This enables more tailored care planning and efficient resource allocation, especially for vulnerable elderly populations. Future research should focus on validating these cutoffs in diverse populations and healthcare settings to foster broader clinical adoption and cross-cultural consensus.

## Summary statement of implications for practice

What does the research add to existing knowledge in gerontology?

- Establishes, for the first time, a quantitative conversion system between the Chinese scale (LS) and the international standard (BI).
- Reveals the effect of age stratification, showing that the corresponding BI scores for LS grades vary among different age groups.
- Demonstrates that picture-based LS grades, integrated with the BI cutoffs, enable precise classification, advancing gerontology from a universal single-scale approach to multiple evaluation models.

What are the implications of this new knowledge for nursing care for older people?

- Universal screening: A BI score ≥75 can serve as a reliable indicator to identify individuals with good functional ability and a lower risk of disability.
- Prioritized resource allocation: A BI score <5 is a clear marker for identifying complete dependency, signaling the need for highest-priority care interventions.
- Advanced age assessment: When assessing individuals aged ≥80 years, it is crucial to recognize their higher disability threshold to avoid overlooking potential care needs based on a deceptively moderate BI score.

How could the findings be used to influence policy, practice, research, or education?

- Long-term care insurance: The BI cutoff score (BI < 5 equivalent to LS Grade 6) serves as a decision trigger for long-term care, enabling the implementation of appropriate care measures for older adults.
- Primary healthcare funding: Additional subsidies should be allocated to community centers based on the coordinated BI-LS assessment care framework.
- Senior care facility protocols: Multiple healthcare settings, including community and home-based care, should be standardized based on the BI cutoff value framework.

## Data Availability

The raw data supporting the conclusions of this article will be made available by the authors, without undue reservation.
