# Supplementary figures and images for: Determining optimal Barthel Index cutoff scores for predicting Longshi Scale grades across age groups in stroke patients
Source: Front Aging. 2026 Feb 9;7:1701910. doi: 10.3389/fragi.2026.1701910 (PMC12926473; doi:10.3389/fragi.2026.1701910)

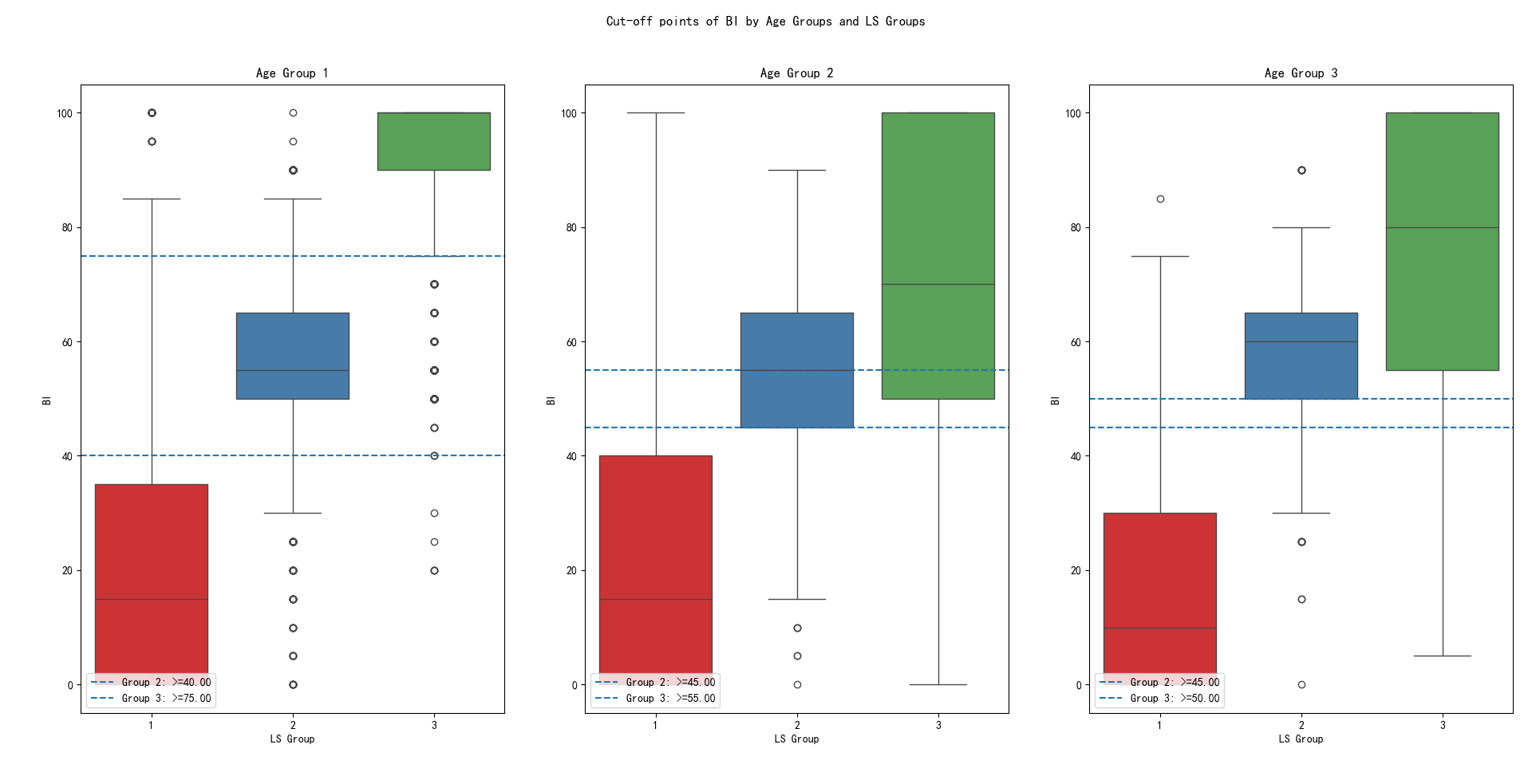

Supplement: Supplementary file 1 [file Image1.tif]

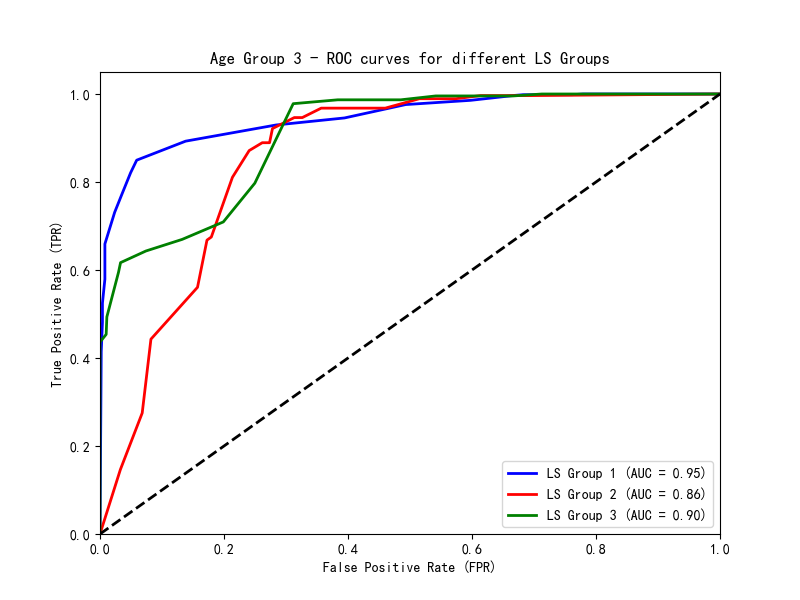

Supplement: Supplementary file 2 [file Image4.png]

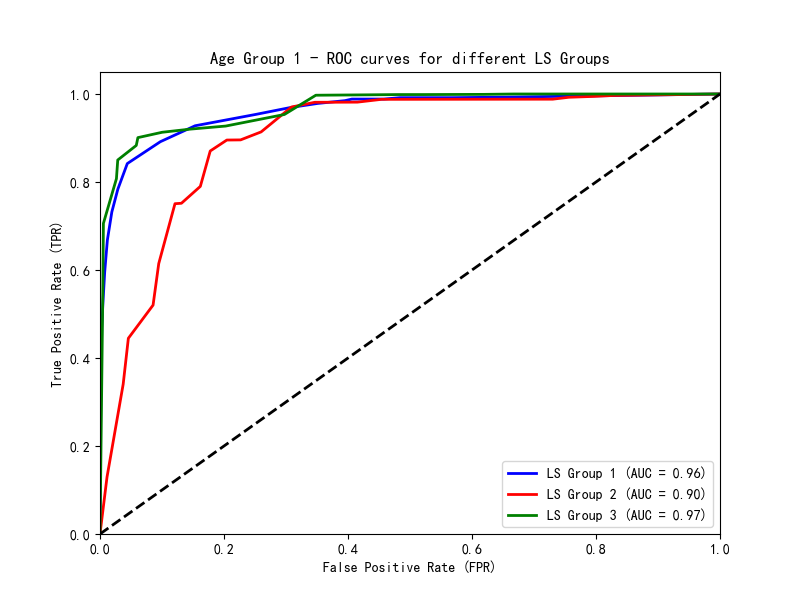

Supplement: Supplementary file 3 [file Image2.png]

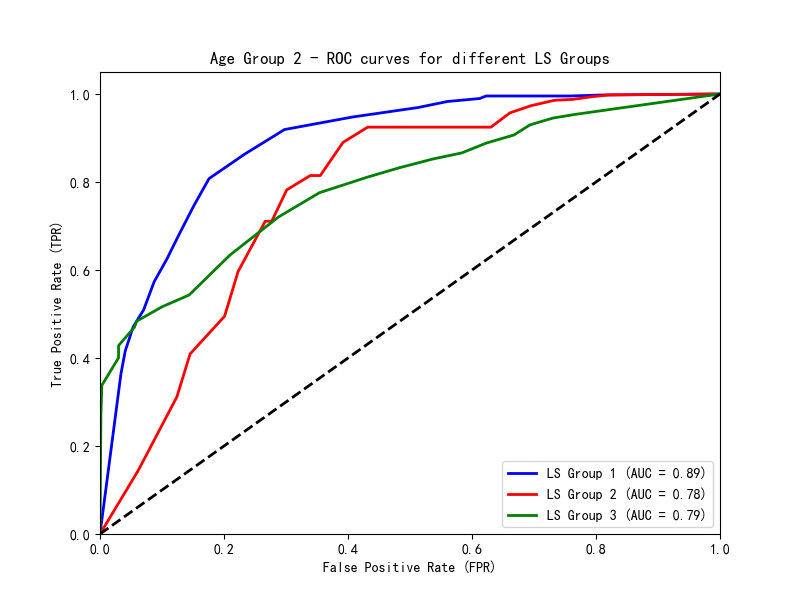

Supplement: Supplementary file 4 [file Image3.png]
